# Sheep feeding preference as a tool to control pine invasion in Patagonia: influence of foliar toughness, terpenoids and resin content

**DOI:** 10.1038/s41598-020-68748-y

**Published:** 2020-07-21

**Authors:** Lucía B. Zamora-Nasca, Romina D. Dimarco, Daniela Nassini, Pablo A. Alvear, Ariel Mayoral, Martin A. Nuñez, M. Andrea Relva

**Affiliations:** 1 Instituto de Investigaciones en Biodiversidad y Medio Ambiente, CONICET - Universidad Nacional del Comahue, Bariloche, Río Negro Argentina; 2 Grupo Ecología de Invasiones. Instituto de Investigaciones en Biodiversidad y Medio Ambiente, CONICET - Universidad Nacional del Comahue, Bariloche, Río Negro Argentina; 3 Departamento de Fisicoquímica y Control de Calidad. Complejo Tecnológico Pilcaniyeu, CNEA, Bariloche, Río Negro Argentina; 4 Grupo de Ecología de Poblaciones de Insectos, IFAB (INTA – CONICET), Modesta Victoria 4450, 8400 Bariloche, Rio Negro Argentina

**Keywords:** Ecology, Invasive species

## Abstract

Herbivores modulate the structure and composition of plant communities, including plant invasions. This is conditioned by plant palatability which can be reduced by its chemical or physical traits. The effects that ungulates browsing has on pine invasions are variable and the empirical evidence on the causes of this variability is scarce. We experimentally explored how sheep browsing preference varies between seedlings of pine species with different invasiveness; *Pinus contorta* (high invasiveness), *P. ponderosa* (medium invasiveness), *P. radiata* (medium invasiveness) and *P. jeffreyi* (low invasiveness). Secondly, we quantified anti-herbivory chemical compounds and physical traits of these species and related them with sheep preference observed. The browsing incidence of *P. contorta* was 68%, *P. ponderosa* 58%, *P. radiata* 29%, and *P. jeffreyi* 84%. Among anti-herbivory traits analyzed, α-pinene concentration had a negative effect on the probability of a terminal bud being browsed and on browsing intensity. Meanwhile, foliar toughness was negatively related to browsing intensity and water concentration was positively related to browsing intensity. Also, the most invasive species, *P. contorta*, was highly damaged. Thus, sheep herbivory could be slowing pine invasion rate; suggesting that could be considered a tool to control early invasions, especially for this particular species.

## Introduction

Herbivory is a strong modulator of the structure and dynamic of the plant community, both for its direct and indirect effects on plants (e.g. changes in the chemical and physical composition of the soil)^1, 2^. In particular, the effect of herbivory on plant invasions has been widely debated in the context of the Natural Enemies Hypothesis, Biotic Resistance, and Enemy Release Hypothesis^3–8^. Herbivores can avoid some species, promoting its invasion, or can limit invasion by consuming them^9, 10^. The role of introduced ungulates in facilitating plant invasions has been demonstrated by many studies^11–17^. Nevertheless, the impact of herbivory on the establishment of non-native species depends on many factors, including plant tolerance, resistance, and palatability.

In the Southern Hemisphere, the invasion by species of the Pinaceae family is well recorded^18^. The family is native to the northern hemisphere and has been introduced in this region mainly for forestry purposes, but also with the aim of control soil erosion or as ornamentals^19^. However, the invasiveness among pine species differs. It was suggested that three pine traits mainly contribute to increasing their invasive capacity: low seed mass, short juvenile period, and short reproduction intervals with large seed production^20^. However, there could be many other factors behind pine invasions, such as abiotic factors (i.e. climatic factors, wind, light availability), the propagule pressure in the area, the presence of mycorrhizal fungi, the biotic resistance of the community where it is introduced, and the presence of herbivores^13, 21–23^.

The effects of ungulates on pine invasions are variable across studies: some studies suggest that they promoted the invasion^22, 24–26^ and others suggest that they hinder the pine invasion^27–30^. For example, it was proposed that sheep and cows could favor the *Pinus sylvestris* and *Pinus mugo* invasion process in Spain, while *Abies alba* could be controlled by these ungulates in the same areas, possibly, because of its higher sensitivity to herbivory damage^31^. Other studies observed that exotic invasive deer preferred native species over the exotic Pinaceae species *Pseudotsuga menziesii* and *Pinus ponderosa* in Argentina^22, 24^. Lastly, in another study, it was observed that feral horses could have promoted the establishment of *Pinus halepensis* seedlings after a long period of grazing in Argentinean Pampean grasslands^26^. Among the studies that suggest that ungulates could be controlling pine invasions; it was observed that semi-feral cattle and sheep prevent the regeneration of *Pinus nigra* and *Pinus sylvestris* in France^28, 29^. Finally, it was suggested that exotic vertebrates herbivores negatively affected *Pinus radiata* survival in Mediterranean areas of Chile^30^. These opposing results may be due to distinctive characteristics of ungulates; such as its behavior, physiology, habits, and origin; as well as intrinsic characteristics of the plant such as phenology, plant morphology, and palatability^32–36^.

Regarding plant palatability, the plant nutritional quality (e.g. proportion of fiber and water content), its physical characteristics (e.g. leaf toughness and trichomes), and their secondary metabolites (e.g. alkaloids and monoterpenes) are important traits that affect the ungulate preference^32–34, 37^. Particularly for mammalian herbivores, in several studies, the preference between conifer species was related to the type and concentrations of secondary compounds^38–44^. For example, total foliar monoterpenes concentration influenced negatively the browsing behavior of Red deer (*Cervus elaphus*)^38^ and Black-tailed deer (*Odocoileus hemionus columbianus*)^39^. However, chemical defenses vary greatly between different conifer species and even between genotypes within a species, causing palatability to differ^45, 46^.

Among ungulates, it is known that sheep exhibit high plasticity in feeding preference, and even though they are considered grazers herbivores, they have been used to successfully control weeds because they can consume plants with higher fiber content than can other domestic ungulates^47^. Although studies are scarce, it has been suggested that regular sheep browsing could limit the spread of species in the Pinaceae family. Ledgard^48^ observed that in New Zealand, even low stocking rates of sheep reduced the successful establishment of *Pinus contorta* seedlings younger than two years old. However, for efficient control, it is necessary more intensive browsing to impede the establishment of new seedlings^49^. Crozier and Ledgard^50^ assessed the palatability of seven pine species to sheep in New Zealand. They found a gradient of consumption, with *Pinus radiata* and *Pinus contorta*, being the most browsed, and in less degree, *Pinus sylvestris*, *Pinus ponderosa*, *Larix decidua*, *Pseudotsuga menziesii*, and *Pinus nigra*. Thus, differential palatability may hinder the control of certain species^1, 9, 51, 52^. Palatability is a relative concept dependent on plant chemical and physical characteristics and ontogeny^32, 53^. Herbivore preference could also depend on the relative presence of palatable plants in the community and the nutritional needs of the herbivore^54, 55^. For example, Cingolani et al.^56^ observed that characteristics that indicate high structural quality for sheep, were in fact avoided and the authors suggest that sheep select plants for their chemicals compounds also nutritionally necessary at the time of the ingest.

The empirical evidence for the control of pine invasion by sheep is scarce at global level^48^ and nonexistent for Patagonia. In this study, we take an experimental approach to explore the potential of sheep herbivory to control pine invasion. First, we experimentally explored how sheep browsing differs across seedlings of four pine species with varying invasiveness: *P. contorta*, *P. ponderosa*, *P. radiata,* and *P jeffreyi*. Second, we quantified anti-herbivore chemical compounds and physical traits in seedlings of these pine species to understand the factors behind the observed preferences.

## Methods

### Study area

This research was conducted in NW Patagonia in the Fortín Chacabuco ranch, located in Neuquén province, Argentina (41° 0′ 16.67″ S and 71° 10′ 46.62″ W). The landscape is mountainous, and it is crossed by many rivers and streams. The climate is Mediterranean, with 60% of precipitation occurring in autumn and winter. The annual rainfall ranges between 300 and 700 mm per year and the average annual temperature does not exceed 10 °C^57^. The vegetation corresponds to a semiarid Patagonian steppe in the Andean piedmont and forms a mosaic depending on exposure and soil. The dominant vegetation is the steppe of tussock grasses, *Pappostipa speciosa* in the lower sectors, and *Festuca pallescens* in higher areas, with scattered shrubs of *Acaena splendens*, *Senecio bracteolatus*, and *Mulinum spinosum*. There are many flood meadows with *Juncus balticus*, *Poa pratensis,* and *Festuca pallescens* associated with shrub of *Ochetophila trinervis, Discaria chacaye*, *Escallonia virgata*, *Berberis microphylla* and *Maytenus boaria*^57^.

The ranch also contains plantations of *P. contorta* and *P. ponderosa*, and pines have invaded some areas. Many pines that have escaped from plantations show signs of herbivory. Besides domestic herbivores (cattle, sheep, and horses), invasive non-native herbivores are present, including red deer (*Cervus elaphus*) and European hare (*Lepus europaeus*), as well as native herbivores such as guanaco (*Lama guanicoe*).

Even though sheep production in Patagonia has decreased in recent decades, it remains the main agricultural activity in Patagonia, and the main production is the Merino breed^58^. As such, sheep are among the most abundant and influential ungulate herbivores in several Patagonian ecosystem processes, including plant invasions^59, 60^.

### Experimental design

To assess sheep preference between four invasive pine species and to estimate anti-herbivores chemical compounds and physical traits of these pine species; we obtained 480 individuals (120 of each species) from a plant nursery that produces seedlings for forestry purposes. We used 400 individuals to perform the preference experiment and we separated 80 individuals from the same population to assess the anti-herbivory chemical compounds and physical traits. The plant nursery is located near the site where we performed the experiment. The seedlings were younger than two years old. The mean initial height of *P. contorta* seedlings was 6.8 cm (SD 2.4), of *P. ponderosa* 9.4 cm (SD 2.4), of *P. radiata* 12.9 cm (SD 3.9) and of *P. jeffreyi* 10.6 cm (SD 2.5).

### Sheep browsing preference

To evaluate the differential preference of sheep (*Ovis aries*) among non-native pines with different invasive capacity, we exposed seedlings of four species to Merino wethers herbivory (castrated male sheep) under a recommended stocking rate on that site. Pine species were *P. contorta* (high invasiveness), *P. ponderosa* and *P. radiata* (moderate invasiveness) and *P. jeffreyi* (low invasiveness).

Within the study area, we installed five experimental enclosures of 25 × 25 m with a 1.50 m tall fence (each enclosure constituted a replicate). Enclosures had hardware cloth 0.6 m tall buried in the soil to prevent access by other herbivores, such as European hare (*L. europaeus*). Each enclosure was stocked with sheep at a density typically recommended for the study area (0.2–0.3 wethers/ha/year for grassland)^61, 62^. This density was based on the assessment of the forage condition of the sites and the area of the enclosure, according to this, the density estimated corresponded to two wethers in each enclosure for three days. More details about the estimation of the stocking rate can be found as Supplementary Methods S1 online. The experiment was conducted in October 2015, during the austral spring, the season with the maximum availability and quality of forage in the community, minimizing the risk of overgrazing. Also, we established the enclosures adjacent to each other, and no sheep were alone, so as not to alter their gregarious behavior. A similar design was used successfully in previous studies in the region^49, 60^. Environmental conditions and forage availability between enclosures corresponding to a replicate were similar. In each enclosure (a replicate) we planted 20 seedlings of each pine species (100 individuals of each species in total), alternating species so that there were not two consecutive individuals of the same species.

The number of browsed seedlings, maximum seedling height before and after the treatment, number of terminal buds damaged, number of browsed branches, the number of defoliated seedlings (i.e. whether the seedling was only the needles damaged, not branches) and the probability of seedling survival immediately after the treatment were recorded for all individuals (100 individuals of each of the four species). The probability of seedling survival was estimated based on the conservative assumption that only seedlings with all branches browsed would die. All seedlings were removed at the end of the experiment.

Because our research was conducted on non-regulated animals in our country, we did not require any ethical approval. However is important to mention that all animals were treated under general animal welfare criteria following the guidelines of National Institutes of Animal production^63, 64^, with constant clean water and food availability (besides the pine seedlings presented during the experiment).

### Anti-herbivores chemical compounds and physical traits

To examine whether seedlings of the four *Pinus* species studied (*Pinus contorta*, *P. ponderosa*, *P. radiata,* and *P. jeffreyi*) differ in their chemical defenses, physical characteristics and plant quality; we measure toughness and monoterpene content in needles of 20 seedlings per species, and 15 out of those 20 seedlings were used to measured non-volatile resin and water content of the needles and branches. It was not possible to perform the sheep preference experiment and these measurements on the same individuals for two reasons. Firstly, since we were studying seedlings (not juveniles or adults), the amount of biomass left after sheep browsed was not enough to measure the chemical defenses and physical traits in the same individual. Secondly, it is known that pine trees have induced defenses^65, 66^, so if we measured the chemistry of the seedling after the sheep fed on them, the chemistry would have changed in response to the damage, and we wanted to relate the browsing preference of sheep to the chemistry and physical traits for the four pine species to tissues that were not previously damaged.

To measure needle toughness, 60 fresh needles from 20 seedlings per *Pinus* species were removed. Needle toughness (g/cm^2^) was measured with a force gauge penetrometer (type 516; Chatillon, Largo, FL, USA) and three measurements were averaged per seedling. The remaining plant material was placed individually in plastic bags and stored in a − 80 ºC freezer for later analyses. For monoterpene extraction, needles were ground with liquid nitrogen in a chilled mortar and pestle to minimize the possible loss of monoterpene. For each sample, the frozen needle powder was put in a separate borosilicate glass vial, and weighted exact weights (between 0.5 and 0.6 g). After recording the weights, 2 mL of GCgrade n-hexane (Merck, Germany) with 0.1 μL mL-1 ( +)-fenchone (Sigma-Aldrich) as an internal standard was added to each vial. Vials were immediately closed with bakelite caps and parafilm to avoid evaporation and mixed with a vortex. The ground needles were left to soak for 7 days at ambient temperature. After a week, 2 mL aliquots of each sample were placed into a clear chromatography autosampler vials for GC analysis. A 1 μL subsample of each aliquot was then injected on a PerkinElmer Clarus 680 Gas Chromatograph with PerkinElmer Clarus 600 T Mass Spectrometer (GC–MS) fitted with an Elite-5 MS column (60 m × 0.25 mm × 0.25 μm; Perkin Elmer) and coupled with a mass spectrometer (PerkinElmer Clarus 600 T Mass Spectrometer) used as detector. The carrier gas used was Helium at a flow rate of 1 mL/min for 20 mL/min split mode. The injector temperature was set at 250 °C to volatilize the liquid sample and the time–temperature history of the oven enclosing the column consisted of three steps: an initial heating at 60 °C during 1 min, a temperature ramp of at 5 °C/min from 60 to 130 °C, and a final holding at 130 °C during 15 min. We could identify four monoterpenes (α-pinene, β-pinene, 3-carene and β-phellandrene) by comparing retention times of known standards and mass spectra using the NIST Mass Spectral Search Program for the NIST/EPA/NIH Mass Spectral Library (version 2.0f) and TurboMass V 5.4.2 software to calculate the area under the absorbance curve for each peak. Since we were unable to obtain a pure standard of the monoterpene β-phellandrene, this monoterpene was measured using the α-phellandrene pure standard being α and β-phellandrenes similar in retention times and mass spectra. Concentrations for each compound were calculated using 9-point calibration curves with injections of known amounts of pure standards and fenchone (the internal standard). All standards were purchased from Sigma Aldrich (Saint Louis, MO).

In order to measure the non-volatile resin content, needles and branches were analyzed together. We cut 15 cm long branches with its needles in pieces, weighted to the nearest 0.0001 g and placed in labeled borosilicate glass tubes. Then each tube was filled with hexane until the material was covered, mixed with a vortex and placed in an ultrasonic bath at 45 °C for 20 min. The tubes were closed and the plant material soaked for 24 h at room temperature. The entire extraction was repeated twice to maximize extraction outputs. The solution was then filtered with paper filters in pre-weighted borosilicate glass tubes and left to evaporate under the fume hood. Each tube has a residual content that corresponded to the non-volatile resin and was weighted to the nearest 0.0001 g. Since non-volatile resin content was obtained from wet weights, a separate subset of 15 cm branches from each sample was dried (oven dry 60 °C until constant weight) and weighed to obtain dry mass conversion factors. The dry weight (DW) of the sample was calculated using the wet weights of the samples and multiplied by each conversion factor. The resin content of the sample was expressed as mg of non-volatile resin /g of stem DW. To assess plant quality, percent water content was calculated as [(wet weight − dry weight)/wet weight] × 100, from the same 15 cm long branches used to measure total non-volatile resin content.

### Data analysis

#### Sheep browsing preference

We used generalized linear mixed models (GLMM)^67, 68^ to test the browsing preference of sheep for four species of pines. The models were fitted with the enclosure as a random effect to control for the possible variability between replicates in adjacent enclosures. The predictor variable was pine species, a factor with four levels. We modeled the following six response variables: (a) the probability of a seedling being browsed (browsing incidence: number of browsed seedlings/total number of seedlings), for this variable, we consider that a seedling was browsed if the terminal bud, and/or any branches, and/or the needles were damaged, (b) the relative reduction in height (initial height − final height/initial height), (c) the probability of a terminal bud being browsed, (d) the proportion of browsed branches per seedling (browsing intensity: number of browsed branches/total number of branches), (e) the probability of a seedling being defoliated (whether only the needles were damaged and it was not damaged the stem or branches), and (f) the probability of seedling survival. Models a, c, d, e, and f were fitted with a *logit* link function and binomial error distribution and model b was fitted with a *logit* link function and beta error distribution.

Because in the preference trial the four species were presented to sheep at the same time, we performed post-hoc comparisons that compared one species with the other three in turn and not a paired comparison. For that analysis, the lsmeans function from “lsmeans” R package^69^ was applied and the “Scheffe” adjust method was used.

The function glmer from the R package “lme4”^70^ was used to fit the browsing incidence, the probability of a terminal bud being browsed, the browsing intensity, the probability of a seedling being defoliated, and the probability of seedling survival models. The function glmmadmb from the “glmmADMB” package^71^ was used to fit the relative reduction in height (initial height − final height/initial height) model.

#### Anti-herbivores chemical compounds and physical traits

To identify differences in total monoterpene between species (that follow a normal distribution) we performed an ANOVA and pairwise post-hoc comparisons of the coefficients were tested using the function glht from the “multcomp” package^72^. The variables α-pinene, β-pinene, 3-carene, β-phellandrene, resins, toughness, and water content did not follow a normal distribution (Shapiro–Wilks Normality Test) so, to test differences in these variables between species, the non- parametric Kruskal–Wallis test was applied, and pairwise post-hoc comparisons of the coefficients were tested using the function kruskalmc from the “pgirmess” R package^73^.

#### Relation between sheep preference and pines’ anti-herbivores chemical compounds and physical traits

With the aim to have an overview of the relation between sheep herbivory and the anti-herbivores compounds assessed we explore two aspects of the experiments detailed before. First, to understand how anti-herbivore traits drive the first approach of sheep and pines (i.e. as browsing repellents), we assessed the relation between the concentrations of chemical compounds and physical traits with the probability of a sheep browse only the terminal bud of the seedlings. On the other hand, to understand how anti-herbivore traits drive sheep browsing intensity on pines (i.e. sheep continues to eat the pine after the first approach), we assessed the relationship between these anti-herbivore defenses and browsing intensity. To identify the relation between the chemical and physical variables (predictor variables) with the probability of a terminal bud being browsed or the browsing intensity (response variables) we performed three generalized linear mixed models (GLMM) for each response variable: (1) total monoterpenes and total resins as predictor variables; (2) monoterpenes separately, i.e. α-pinene, β-pinene, 3-carene and β-phellandrene as predictor variables and (3) toughness and water content as predictor variables. The models were fitted with a *logit* link function and binomial error distribution. Before performing these regressions we checked for collinearity and, following Dormann et al.^74^, we only include variables with a correlation <|0.7|. Only β-pinene and 3-carene were highly correlated (0.77), so for model 2, we do not include β-pinene. Because the number of individuals tested for sheep preferences (20 seedlings of each species in five enclosures, 100 seedlings of each species in total) was greater than individuals tested for chemical and physical content (15 seedlings of each species to test resins and water, and 20 seedlings of each species to test monoterpenes and foliar toughness), we performed the regressions considering the median of each predictor variable in relation to the individuals tested in the preference experiments. We are aware that this analysis has limitations, but it is appropriate to give a general idea of the principal relations between the anti-herbivory compounds and the sheep preference observed. Also, this approach is used in other biochemical studies due to the high cost and difficulties to assess these traits in larger sampler sizes^75^. Predictor variables were standardized. The models were nested, fitting species as random effects to consider the possible variability between them. In the cases where there was no variation between species, we fitted a generalized linear model (GLM) without random effects. The function glmer from the R package “lme4”^70^ was used. Because the predictor variables were standardized, the model estimates correspond to the magnitude of change in the response variable if the predictor variable changes by one deviation standard. Inferences were made from parameters with *P* < 0.05. The R package ggplot2^76^ was used for visualization of the models. R version 3.5.0 was used^77^.

## Results

### Sheep browsing preference

**Figure 1:**
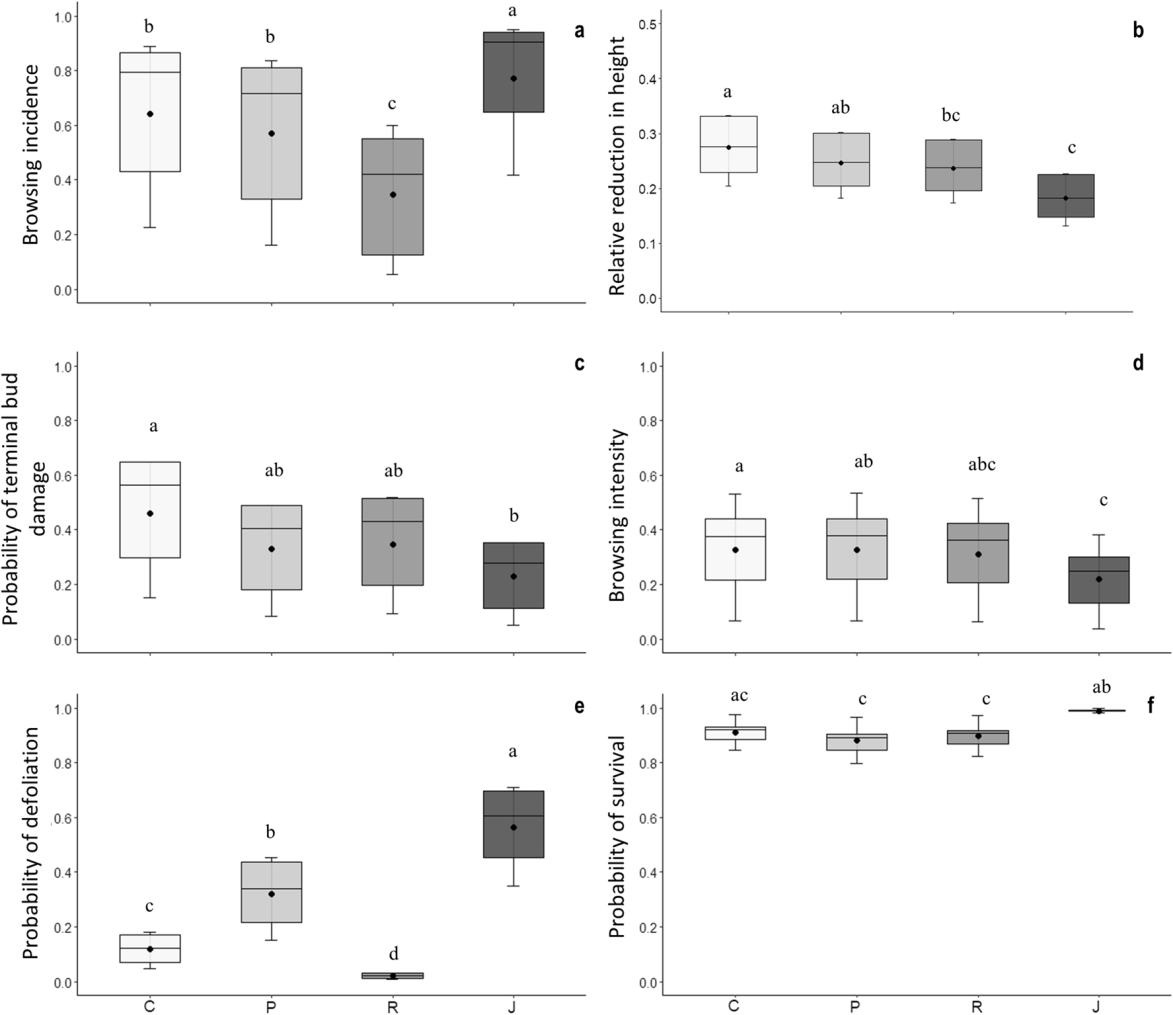
Herbivory on four pine species with different invasiveness: C: *P. contorta* (high invasiveness), P: *P. ponderosa* and R: *P. radiata* (moderate invasiveness), and J: *P. jeffreyi* (low invasiveness). (**a**) Estimated probability of browsing incidence. (**b**) Relative reduction in seedling height. (**c**) Estimated probability of a terminal bud being browsed. (**d**) Estimated probability of browsing intensity. (**e**) Estimated probability of a seedling being defoliated (only the needles damaged). (**f**) Estimated probability of seedling survival. Black point refers to the mean and black line to the median, whiskers correspond to SE. Different letters above boxplots indicate significant differences (Scheffe method).

In general terms, the browsing incidence on *P. contorta, P. ponderosa, P. radiata,* and *P. jeffreyi* seedlings, as estimated by the model, was 68%, 58%, 29%, and 84% respectively (Table 1). *Pinus radiata* seedlings were significantly less browsed than the seedlings of the other three pine species, while *P. jeffreyi* seedlings were significantly more browsed than the seedlings of the other species (Fig. 1a, results of post-hoc comparisons can be found as Supplementary Table S2.1 online).

**Table 1:** Results of the models performed to analyze sheep herbivory preference between four invasive pine species.

Model	Coefficient	Estimate	SE	z value	Pr(>|z|)	Probability	95% CI	
							Lower	Upper
Browsing incidence	Intercept	0.76	0.65	1.17	0.2412	0.68	0.32	0.91
	*P. jeffreyi*	0.89	0.38	2.37	**0.0179**	0.84	0.53	0.96
	*P. ponderosa*	− 0.43	0.35	− 1.22	0.2215	0.58	0.23	0.86
	*P. radiata*	− 1.67	0.36	− 4.61	**3.95E**−**06**	0.29	0.08	0.64
Relative reduction in seedling height	Intercept	− 0.99	0.18	− 5.39	**7.20E**−**08**	0.27	0.20	0.35
	*P. jeffreyi*	− 0.53	0.17	− 3.13	**0.0018**	0.18	0.13	0.24
	*P. ponderosa*	− 0.15	0.17	− 0.85	0.3957	0.24	0.18	0.31
	*P. radiata*	− 0.2	0.17	− 1.17	0.2432	0.23	0.17	0.3
Probability of terminal bud damage	Intercept	− 0.24	0.49	− 0.48	0.628751	0.44	0.2	0.71
	*P. jeffreyi*	− 1.22	0.34	− 3.62	**0.000291**	0.19	0.06	0.42
	*P. ponderosa*	− 0.65	0.32	− 2.04	**0.041353**	0.29	0.11	0.56
	*P. radiata*	− 0.54	0.32	− 1.71	0.087385	0.31	0.12	0.59
Browsing intensity	Intercept	− 0.92	0.46	− 1.99	**0.0466**	0.28	0.11	0.54
	*P. jeffreyi*	− 0.6	0.14	− 4.14	**3.45E**−**05**	0.18	0.07	0.4
	*P. ponderosa*	0.01	0.18	0.05	0.9566	0.29	0.11	0.55
	*P. radiata*	− 0.07	0.24	− 0.27	0.7846	0.27	0.1	0.54
Probability of defoliation	Intercept	− 2.15	0.44	− 4.93	**8.18E**−**07**	0.1	0.04	0.22
	*P. jeffreyi*	2.41	0.39	6.25	**4.23E**−**10**	0.56	0.36	0.75
	*P. ponderosa*	1.31	0.39	3.41	**0.000656**	0.3	0.15	0.5
	*P. radiata*	− 1.91	0.78	− 2.45	**0.014239**	0.017	0.002	0.06
Probability of survival	Intercept	2.53	0.52	4.84	**1.30E**−**06**	0.93	0.81	0.98
	*P. jeffreyi*	2.31	1.05	2.21	**0.0274**	0.99	0.96	1
	*P. ponderosa*	− 0.33	0.47	− 0.71	0.4753	0.9	0.76	0.97
	*P. radiata*	− 0.16	0.48	− 0.33	0.74	0.91	0.79	0.98

We present the estimated value (*Logit* scale), standard error, z-value and their associated *p *value, and probability and lower and upper limits of the 95% confidence interval. Significant *p* values are highlighted in bold.

The relative reduction in *P. contorta, P. ponderosa, P. radiata,* and *P. jeffreyi* seedling height estimated by the model was 27%, 24%, 23%, and 18% respectively (Table 1) with the reduction in height of *P. contorta* seedlings significantly higher than the reductions in height of *P. radiata* and *P. jeffreyi* seedlings; and reductions in height of *P. ponderosa* seedlings were significantly greater than the reduction in height of *P. jeffreyi* seedlings (Fig. 1b; results of post-hoc comparisons can be found as Supplementary Table S2.1 online).

The probability that a terminal bud of a *P. contorta*, a *P. ponderosa*, a *P. radiata* and a *P. jeffreyi* seedling being damaged, as estimated by the model, was 44%, 29%, 31%, and 19% respectively (Table 1); with the proportion of terminal bud browsed of *P. contorta* seedlings significantly higher than the proportion of terminal bud browsed of *P. jeffreyi* (Fig. 1c, results of post-hoc comparisons can be found as Supplementary Table S2.1 online).

The browsing intensity of *P. contorta, P. ponderosa, P. radiata* and *P. jeffreyi* seedlings estimated by the model was 28%, 29%, 27%, and 18%, respectively (Table 1); with the browsing intensity of *P. jeffreyi* significantly lower than the browsing intensity of *P. contorta* and *P. ponderosa* (Fig. 1d, results of post-hoc comparisons can be found as Supplementary Table S2.1 online).

In terms of the probability of a seedling having only the needles damaged, i.e., being defoliated, the probability estimated by the model for *P. contorta, P. ponderosa, P. radiata* and *P. jeffreyi* seedling was 10%, 30%, 1.7%, and 56% respectively (Table 1). These percentages differed significantly for the four species (Fig. 1e, results of post-hoc comparisons can be found as Supplementary Table S2.1 online).

The probability of survival of a *P. contorta,* a *P. ponderosa,* a *P. radiata* and a *P. jeffreyi* seedling immediately after the treatment was 93%, 90%, 91% and 99% respectively (Table 1), with the probability of survival of *P. jeffreyi* significantly higher than the probability of survival of *P. ponderosa* and *P. radiata* seedlings (Fig. 1f; results of post-hoc comparisons can be found as Supplementary Table S2.1 online).

### Anti-herbivores chemical compounds and physical traits

**Figure 2:**
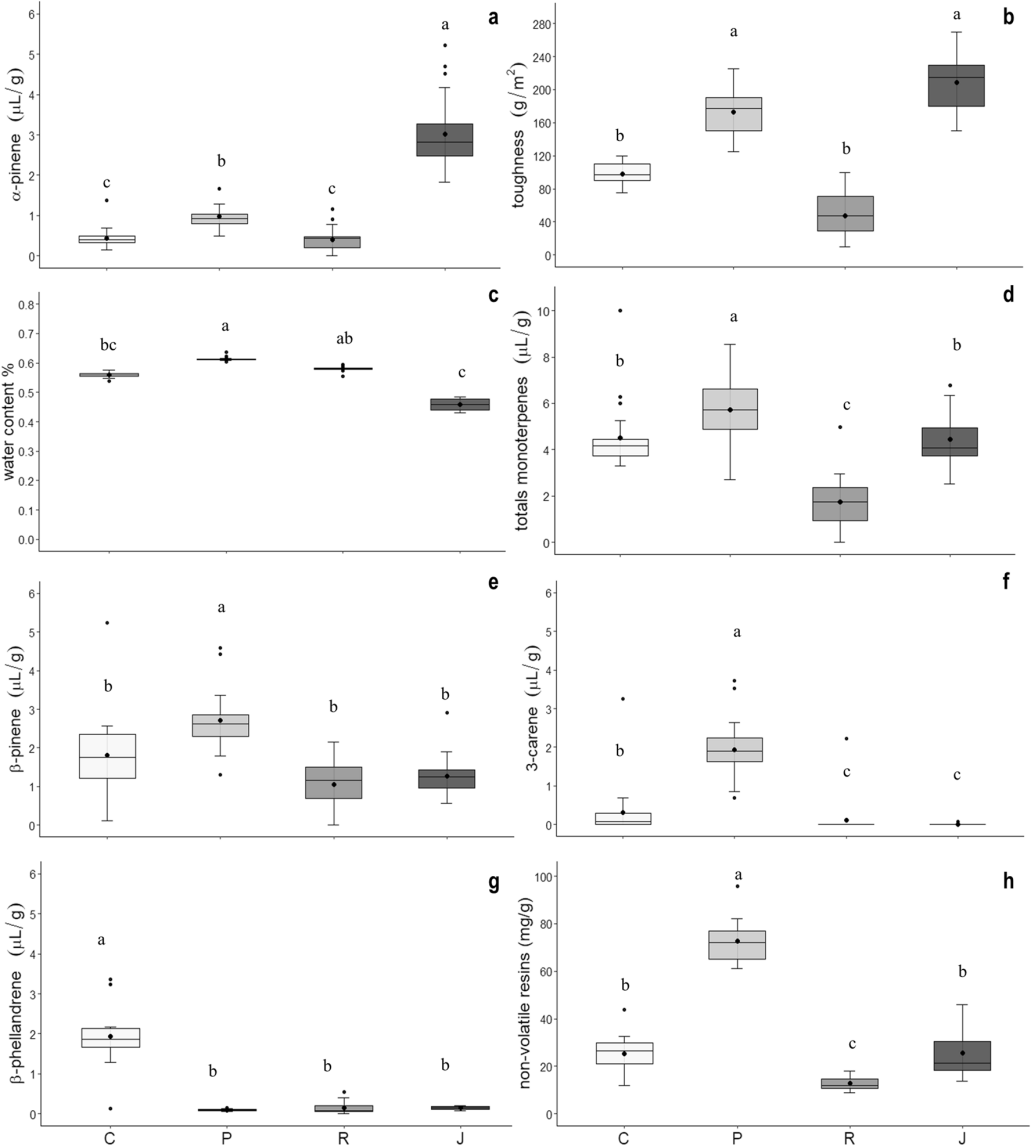
Chemical compounds and physical traits of four pine species with different invasiveness: C: *P. contorta* (high invasiveness), P: *P. ponderosa* and R: *P. radiata* (moderate invasiveness), and J: *P. jeffreyi* (low invasiveness). (**a**) α-pinene, (**b**) Toughness, (**c**) Water content, (**d**) Total monoterpenes, (**e**) β-pinene, (**f**) 3-carene, (**g**) β-phellandrene, (**h**) non-volatile resins. Black point refers to the mean and black line to the median, whiskers correspond to SE. Different letters above boxplots indicate significant differences. Data of total monoterpenes, α-pinene β-pinene, β-phellandrene, 3-carene, and toughness was taken from the needles and data of non-volatile resins content and water content was taken from branches and needles together.

The mean concentration of α-pinene in seedlings of *P. contorta, P. ponderosa, P. radiata,* and *P. jeffreyi* was 0.44, 0.98, 0.40 and 3.02 μL/g respectively; with the concentration of *P jeffreyi* seedlings significantly higher than the other three species, and the concentration of *P. ponderosa* seedlings significantly higher than the concentration of *P. contorta* and *P. radiata* seedlings (Kruskal–Wallis chi-squared = 61.177, *df* = 3, *p* value = 3.294e−13; Fig. 2a, results of post-hoc comparisons can be found as Supplementary Table S3.2 online).

The mean foliar toughness of *P. contorta, P. ponderosa, P. radiata* and *P. jeffreyi* seedlings was 98.25, 173.25, 47.75 and 209 g/cm^2^ respectively; with the toughness of *P. ponderosa* and *P. jeffreyi* significantly higher than the toughness of *P. contorta* and *P. radiata* seedlings (Kruskal–Wallis chi-squared = 67.654, *df* = 3, *p* value = 1.357e−14; Fig. 2b, results of post-hoc comparisons can be found as Supplementary Table S3.2 online).

The percent water content of *P. contorta, P. ponderosa, P. radiata* and *P. jeffreyi* seedlings was 0.56, 0.61, 0.58 and 0.46% respectively; with the percent water content of *P. ponderosa* significantly higher than the percent water content of *P. contorta* and *P. jeffreyi* and the percent water content of *P. radiata* significantly higher than the percent water content of *P. jeffreyi* (Kruskal–Wallis chi-squared = 54.388, *df* = 3, *p* value = 9.274e−12; Fig. 2c, results of post-hoc comparisons can be found as Supplementary Table S3.2 online).

The concentrations of total monoterpenes, β-pinene, 3-carene, β-phellandrene, and non-volatile resins differed between pine species (Fig. 2d, e, f, g, and h, respectively). Mean values and results of statistical analyses are detailed in Tables 2 and 3 (Results of post-hoc comparisons can be found as Supplementary Table S3.2 online).

**Table 2:** Mean concentration of totals Monoterpenes in four invasive species of pine and results of ANOVA.

	Mean concentration per species				ANOVA results		
	*P. contorta*	*P. ponderosa*	*P. radiata*	*P. jeffreyi*	F	*df*	*p*
Total monoterpenes (μL/g)	4.51	5.72	1.73	4.45	29.35	3	**1.04e**−**12**

**Table 3:** Mean concentration of chemical anti-herbivory compounds in four invasive species of pine and results of Kruskal–Wallis test.

	Mean concentration per species				Kruskal–Wallis test results		
	*P. contorta*	*P. ponderosa*	*P. radiata*	*P. jeffreyi*	X^2^	*df*	*p*
β-Pinene (μL/g)	1.81	2.7	1.06	1.26	36.52	3	**5.81E−08**
β-Phellandrene (μL/g)	1.94	0.1	0.15	0.15	47.61	3	**2.58E−10**
3-Carene (μL/g)	0.30	1.94	0.11	0.01	56.83	3	**2.791e−12**
Non-volatile resins (mg/g DW)	25.33	72.72	12.8	25.73	46.87	3	**3.705e−10**

### Relation between sheep preference and pines’ anti-herbivores chemical compounds and physical traits

Here, we report the general inferences made from parameters with *p* < 0.05. Details about final models, parameter estimates, significances, and confidence intervals can be found in Tables 4 and 5. Out of all anti-herbivores defenses analyzed, only α-pinene had a significant negative effect on the probability of a terminal bud being browsed (Table 4). The concentrations of α-pinene and foliar toughness were negatively related to browsing intensity (Table 5). Conversely, water concentration was positively related to browsing intensity (Table 5).

**Table 4:** Results of models that assess the probability of a terminal bud being browsed in relation to chemical compounds and physical traits of pine seedlings.

Model	Coefficients	Estimate	SE	Z value	95% CI		Pr(>|z|)
					Lower	Upper	
M1	α (Intercept)	− 0.67	0.18	− 3.63	− 1.15	− 0.21	**0.0002**
	β_1_ (total monoterpenes)	− 0.08	0.36	− 0.21	− 1.00	0.82	0.833
	β_2_ (resins)	0.07	0.36	0.18	− 0.83	1.00	0.854
M2	α (Intercept)	− 0.68	0.11	− 6.27	− 0.89	− 0.47	**3.7e−10**
	β_1_ (α-pinene)	− 0.34	0.12	− 2.91	− 0.57	− 0.12	**0.004**
M3	α (Intercept)	− 0.67	0.13	− 5.20	− 1.00	− 0.36	**1.99e−07**
	β_1_ (toughness)	− 0.19	0.15	− 1.29	− 0.56	0.18	0.197
	β_2_ (water)	0.13	0.15	0.85	− 0.24	0.51	0.397

We present the estimated value (*Logit* scale), standard error, z-value and their associated *p *value, and the lower and upper limits of the 95% confidence interval. Significant *p *values are highlighted in bold. M1: Results of the model that assess the probability of a terminal bud being browsed in relation to total monoterpenes and total resins concentration. α is the baseline probability, β_1_ is the regression coefficient that represents the effects of totals monoterpenes; β_2_ is the regression coefficient that represents the effects of totals resin content. M2: Results of the model that assess the probability of a terminal bud being browsed in relation to α-pinene concentration. α is the baseline probability, β_1_ is the regression coefficient that represents the effects of α-pinene concentration. M3: Results of the model that assess the probability of a terminal bud being browsed in relation to foliar toughness and water content. α is the baseline probability, β_1_ is the regression coefficient that represents the effects of foliar toughness; β_2_ is the regression coefficient that represents the effects of water content.

**Table 5:** Results of models that assess the browsing intensity in relation to chemical compounds and physical traits of pine seedlings.

Model	Coefficients	Estimate	SE	Z value	95% CI		Pr(>|z|)
					Lower	Upper	
M1	α (Intercept)	− 0.87	0.14	− 6.09	− 1.23	− 0.53	**1.17e**−**09**
	β_1_ (total monoterpenes)	− 0.19	0.29	− 0.68	− 0.89	0.50	0.497
	β_2_ (resins)	0.25	0.27	0.91	− 0.43	0.93	0.362
M2	α (Intercept)	− 0.84	0.05	− 14.24	− 0.96	− 0.73	**< 2e**−**16**
	β_1_ (α-pinene)	− 0.29	0.05	− 5.71	− 0.40	− 0.20	**1.11e**−**08**
M3	α (Intercept)	− 0.81	0.06	− 13.28	− 0.93	− 0.69	**< 2e**−**16**
	β_1_ (toughness)	− 0.16	0.08	− 1.98	− 0.33	0.00	**0.04802**
	β_2_ (water)	0.19	0.07	2.61	0.05	0.33	**0.00903**

We present the estimated value (*Logit* scale), standard error, z-value and their associated *p *value, and the lower and upper limits of the 95% confidence interval. Significant *p *values are highlighted in bold. M1: Results of the model that assess the probability of browsing intensity in relation to total monoterpenes and total resins concentration. α is the baseline probability, β_1_ is the regression coefficient that represents the effects of totals monoterpenes; β_2_ is the regression coefficient that represents the effects of totals resin content. M2: Results of the model that assess the probability of browsing intensity in relation to α-pinene concentration. α is the baseline probability, β_1_ is the regression coefficient that represents the effects of α-pinene concentration. M3: Results of the model that assess the probability of browsing intensity in relation to foliar toughness and water content. α is the baseline probability, β_1_ is the regression coefficient that represents the effects of foliar toughness; β_2_ is the regression coefficient that represents the effects of water content.

## Discussion

We show that all pine species were browsed by sheep; however, the magnitude and the pattern of damage differed. Regarding the browsing incidence, *P. jeffreyi* was the most preferred species, followed by *P. contorta*, and *P. ponderosa*, and lastly *P. radiata*. That is, sheep mostly preferred the least invasive and the most invasive species. But, regarding browsing intensity, sheep browsed more intensely the most invasive pine species. Since the four species were damaged, sheep browsing at this stocking rate could be affecting the growth rate and retarding seedlings from growing into adults^78^, slowing down the invasion rate, although not stopping it.

Regarding the chemical compounds, we observed that the total concentration of monoterpenes does not influence the attractiveness of pines to sheep, nor the subsequent damage as we did not find a significant effect on the probability of browse on terminal buds not browsing intensity. But looking specifically across monoterpenes, the concentration of α-pinene was the major deterrent to browsing. Therefore, in general terms, the concentration of total monoterpenes does not prevent the browsing of pines by sheep but the presence of certain types of terpenes could be conditioning their pattern of herbivory. The negative effects of α-pinene concentration on sheep browsing observed in our study were also observed in another study^79^. Estell et al.^79^ performed an experiment assessing intake by sheep of alfalfa pellets treated with six chemical compounds (camphor, limonene, *cis*-jasmone, β-caryophyllene, borneol, or α-pinene), each one at five different concentrations. They observed a negative effect of chemical concentration on the intake of camphor and α-pinene. The general lack of anti-herbivore effect by monoterpenes concentration (except α-pinene) observed in our study could be due to the sheep domestic condition, probably individuals with high reaction to adverse stimuli were not selected during domestication^80–82^.

We found that foliar toughness negatively influenced browsing intensity. The differential browsing observed between the four species (terminal bud, lateral branches or needle damage) may be due to the differences in morphology and/or degree of lignification of each species (all seedlings were younger than two years old, thus these differences were not due to age, but rather intrinsic differences of the species). *Pinus jeffreyi* and *P. ponderosa* seedlings presented the highest foliar toughness and also, they were the most defoliated. Cingolani et al.^56^ measured plant functional traits and estimated sheep selectivity and plant grazing response, they observed that characteristics that indicate high structural quality for herbivores, between them low toughness, were in fact avoided suggesting that chemical defenses could be playing a more important role for this herbivore. In fact, Villalba and Provenza^83^ posed the hypothesis that food structure and nutritional composition interact to determine the lambs' preferences, so, when a particular nutrient is needed, the biochemical composition is more important than the structure in determining the preference. This pattern was also observed by Evju et al.^84^ in a study of sheep selectivity, they found that sheep select large, late-flowering herbs that have higher nutritional qualities (low leaf C/N ratio). In our study, *P. jeffreyi* and *P. ponderosa*, species with high browsing incidence, could have higher nutritional qualities for sheep, even when the physical structure was not the ideal for a grazer herbivore (high toughness). Regarding nutritional qualities, in our study, we only assessed seedlings’ water content. We observed that this variable had a significant positive influence on browsing intensity. It would be important to complement this work with studies of the influence of other nutritional compounds on sheep’s preference, like carbon, nitrogen, and phosphorus content.

It is important to clarify that due to the well-known induced response of Pinaceae to herbivory^65, 66^, the preference experiments and the anti-herbivory traits were measured in different plant material. Measuring these different variables in different plants might create differences that are not possible to account for. Even though all the seedlings came from the same source and were from the same pool, it is possible that other factors influenced the preference observed besides the traits assessed in our work, like differential microsite growth condition of each individual or morphological differences that were not possible to includes in the analyses, like plant height. However, we think that the performed analyses were the best possible approach to further our understanding of the relations between the anti-herbivory compounds and the sheep preference observed.

Some studies suggest that plant invasions could be controlled with mammal herbivory in other systems^28–30, 85, 86^. In Patagonia, Sarasola et al.^27^ suggested that domestic ungulates could be one of the factors that are controlling pines invasions. However, in that study, the authors did not detail the species of ungulate, and for Patagonia, no empirical evidence about sheep herbivory on pines has been recorded to date. We observed that sheep damaged pine seedlings according to this decreasing gradient of preference: *Pinus jeffreyi, Pinus contorta, Pinus ponderosa,* and *Pinus radiata.* This suggests that the most invasive species, *P. contorta*, could potentially be controlled with sheep herbivory. In another study in New Zealand, Crozier and Ledgard^50^ found that *Pinus radiata* and *Pinus contorta* were the most consumed species by sheep, followed by *Pinus ponderosa*. Our results correspond partially with this gradient of consumption since *P. radiata* was the least preferred species. In another study done in Puerto Patriada, Argentina, it was found that *P. radiata* individuals from different cohorts growing outside of plantations had varying non-volatile resin content of needles and branches (Dimarco personal communication). The difference in the sheep preference gradient found in our study and the one found by Crozier and Ledgard in New Zealand could be explained by the distinctive levels in chemical defenses that different genotypes of the same plant species could possess^46, 87^, and also by the growing conditions of the pine seedlings used by Crozier and Ledgard and in our study.

Even though *P. contorta* was preferred by sheep in the present study, we observed in a previous study that the stocking rate implemented will determine the effectiveness of the invasion control^49^. The stocking rate must be estimated taking into account the palatability, the pine age, the regeneration rate, and the degree of invasion of the focal species. If the species to control are unpalatable, it could be necessary stock management concentrating high stocking rates in key seasons (i.e. spring)^88^. Regarding the age, previous studies observed that the concentration of anti-herbivores compounds changes with the ontogeny^89^. So, we suggest performing an herbivory invasion control over seedling younger than 2 years old^50^ to guarantee high browse intensity of the seedling in order to avoid branch resprouting and ensure mortality. Lastly, Ledgard^48^, suggested that *P. contorta* is among the planted pine species with the highest regeneration rate. Thus, taking into account all the variables mentioned, for an effective herbivory control of *P. contorta* invasion (one of the most invasive species in the Southern Hemisphere) we suggest apply a high stocking rate in news fronts of invasion during short periods but with an annual frequency.

Our study suggests that sheep browsing could be a practical tool to control pine spread beyond the plantations. We observed that sheep consumed seedlings across our pine species at different intensities. Herbivory pine control could be more effective for pine species with low α-pinene concentration, low foliar toughness, and high nutritional qualities. Forestry practices accompanied by appropriate stock management plans could be an efficient method to control early pine invasions.

## Supplementary information

Supplementary Information 1.

## Data Availability

The datasets generated during the current study are available from the corresponding author upon request.

## References

[1] Augustine DJ, McNaughton SJ (1998). Ungulate effects on the functional species composition of plant communities: herbivore selectivity and plant tolerance. J. Wildl. Manag..

[2] Huntly N (1991). Herbivores and the dynamics of communities and ecosystems. Annu. Rev. Ecol. Syst..

[3] Keane RM, Crawley MJ (2002). Exotic plant invasions and the enemy release hypothesis. Trends Ecol. Evol..

[4] Maron JL, Vila M (2001). When do herbivores affect plant invasion? Evidence for the natural enemies and biotic resistance hypotheses. Oikos.

[5] Lockwood JL, Hoopes MF, Marchetti MP, Lockwood JL, Hoopes MF, Marchetti MP (2013). Establishment success: the influence of biotic interactions. Invasion Ecology.

[6] Meijer K, Schilthuizen M, Beukeboom L, Smit C (2016). A review and meta-analysis of the enemy release hypothesis in plant-herbivorous insect systems. PeerJ.

[7] Nunez-Mir GC (2017). Biotic resistance to exotic invasions: its role in forest ecosystems, confounding artifacts, and future directions. Biol. Invasions.

[8] Jeschke JM, Heger T (2018). Invasion Biology. Hypotheses and Evidence.

[9] Averill KM, Mortensen DA, Smithwick EAH, Post E (2016). Deer feeding selectivity for invasive plants. Biol. Invasions.

[10] Parker J, Burkepile D, Hay M (2006). Opposing effects of native and exotic herbivores on plant invasions. Science.

[11] Hobbs RJ (2001). Synergisms among habitat fragmentation, livestock grazing, and biotic invasions in Southwestern Australia. Conserv. Biol..

[12] Knight TM, Dunn JL, Smith LA, Davis J, Kalisz S (2009). Deer facilitate invasive plant success in a Pennsylvania forest understory. Nat. Areas J..

[13] Nuñez MA (2013). Exotic mammals disperse exotic fungi that promote invasion by exotic trees. PLoS ONE.

[14] Oduor AMO, Gómez JM, Strauss SY (2010). Exotic vertebrate and invertebrate herbivores differ in their impacts on native and exotic plants: a meta-analysis. Biol. Invasions.

[15] Spear D, Chown SL (2009). Non-indigenous ungulates as a threat to biodiversity. J. Zool..

[16] Vavra M, Parks CG, Wisdom MJ (2007). Biodiversity, exotic plant species, and herbivory: the good, the bad, and the ungulate. For. Ecol. Manag..

[17] Loydi A, Zalba SM (2009). Feral horses dung piles as potential invasion windows for alien plant species in natural grasslands. Plant Ecol..

[18] Richardson DM, Rejmánek M (2004). Conifers as invasive aliens: a global survey and predictive framework. Divers. Distrib..

[19] Simberloff D (2010). Spread and impact of introduced conifers in South America: lessons from other southern hemisphere regions. Austral. Ecol..

[20] Rejmánek M, Richardson DM (1996). What attributes make some plant species more invasive?. Ecology.

[21] Nuñez MA, Simberloff D, Relva MA (2008). Seed predation as a barrier to alien conifer invasions. Biol. Invasions.

[22] Nuñez MA, Relva MA, Simberloff D (2008). Enemy release or invasional meltdown? Deer preference for exotic and native trees on Isla Victoria Argentina. Austral. Ecol..

[23] Nuñez MA, Medley KA (2011). Pine invasions: climate predicts invasion success; something else predicts failure. Divers. Distrib..

[24] Relva MA, Nuñez MA, Simberloff D (2010). Introduced deer reduce native plant cover and facilitate invasion of non-native tree species: evidence for invasional meltdown. Biol. Invasions.

[25] Osem Y, Lavi A, Rosenfeld A (2011). Colonization of *Pinus halepensis* in Mediterranean habitats: consequences of afforestation, grazing and fire. Biol. Invasions.

[26] de Villalobos A, Zalba SM, Peláez DV (2011). *Pinus halepensis* invasion in mountain pampean grassland: effects of feral horses grazing on seedling establishment. Environ. Res..

[27] Sarasola MM, Rusch VE, Schlichter TM, Ghersa CM (2006). Invasión de coníferas forestales en áreas de estepa y bosques de ciprés de la cordillera en la Región Andino Patagónica. Ecol. Austral..

[28] Chauchard S, Pille G, Carcaillet C (2006). Large herbivores control the invasive potential of nonnative Austrian black pine in a mixed deciduous Mediterranean forest. Can. J. For. Res..

[29] Boulant N, Kunstler G, Rambal S, Lepart J (2008). Seed supply, drought, and grazing determine spatio-temporal patterns of recruitment for native and introduced invasive pines in grasslands. Divers. Distrib..

[30] Becerra PI, Bustamante RO (2009). The effect of herbivory on seedling survival of the invasive exotic species *Pinus radiata* and *Eucalyptus globulus* in a Mediterranean ecosystem of Central Chile. For. Ecol. Manag..

[31] Bartolomé J, Boada M, Saurí D, Sánchez S, Plaixats J (2008). Conifer dispersion on subalpine pastures in Northeastern Spain: characteristics and implications for rangeland management. Rangel. Ecol. Manag..

[32] Forbes JM, Forbes JM (2007). Learning about food: conditioned preferences and aversions. Voluntary Food Intake and Diet Selection in Farm Animals.

[33] Danell K, Bergström R, Edenius L (1994). Effects of large mammalian browsers on architecture, biomass, and nutrients of woody plants. Source J. Mammal..

[34] McNaughton SJ (1979). Grazing as an optimization process: grass-ungulate relationships in the Serengeti. Am. Nat..

[35] Canham CD, McAninch JB, Wood DM (1994). Effects of the frequency, timing, and intensity of simulated browsing on growth and mortality of tree seedlings. Can. J. For. Res..

[36] Persson IL, Danell K, Bergström R (2005). Different moose densities and accompanied changes in tree morphology and browse production. Ecol. Appl..

[37] Pollock ML, Lee WG, Walker S, Forrester G (2007). Ratite and ungulate preferences for woody New Zealand plants: influence of chemical and physical traits. N. Z. J. Ecol..

[38] Duncan AJ, Hartley SE, Iason GR (1994). The effect of monoterpene concentrations in Sitka Spruce (*Picea sitchensis*) on the browsing behavior of red deer (*Cervus elaphus*). Can. J. Zool. Can. Zool..

[39] Kimball BA, Russell JH, Ott PK (2012). Phytochemical variation within a single plant species influences foraging behavior of deer. Oikos.

[40] Zhang X, States JS (1991). Selective herbivory of Ponderosa pine by Abert squirrels: a re-examination of the role of terpenes. Biochem. Syst. Ecol..

[41] Elliott S, Loudon A (1987). Effects of monoterpene odors on food selection by red deer calves (*Cervus elaphus*). J. Chem. Ecol..

[42] Bryant JP (1991). Interactions between woody plants and browsing mammals mediated by secondary metabolites. Annu. Rev. Ecol. Syst..

[43] Bryant JP, Reichardt PB, Clausen TP (1992). Chemically mediated interactions between woody plants and browsing mammals. J. Range Manag..

[44] Baraza E, Hódar JA, Zamora R (2009). Consequences of plant-chemical diversity for domestic goat food preference in Mediterranean forests. Acta Oecol..

[45] Moreira X (2014). Trade-offs between constitutive and induced defences drive geographical and climatic clines in pine chemical defences. Ecol. Lett..

[46] Radwan MA, Crouch GL (1978). Selected chemical constituents and deer browsing preference of Douglas Fir. J. Chem. Ecol..

[47] Frost RA, Launchbaugh KL (2003). Grazing for Rangeland Weed Managenent: a new look at an old tool. Rangelands.

[48] Ledgard NJ (2001). The spread of lodgepole pine (*Pinus contorta*, Dougl.) in New Zealand. For. Ecol. Manag..

[49] Zamora-Nasca LB, Relva MA, Núñez MA (2018). Ungulates can control tree invasions: experimental evidence from nonnative conifers and sheep herbivory. Biol. Invasions.

[50] Crozier ER, Ledgard NJ, Basset C, Whitehouse LJ, Zabkiewicz JA (1990). Palatability of wilding conifers and control by simulated sheep browsing. Alternatives to the Chemical Control of Weeds. Proceedings of International Conference, Rotorua, July 1989. Bulletin No. 155.

[51] Mayle B (1999). Domestic Stock Grazing to Enhance Woodland Biodiversity.

[52] Westoby M (1999). The LHS Strategy Scheme in Relation to Grazing and Fire. VIth International Rangeland Congress.

[53] Westoby M (1974). An analysis of diet selection by large generalist herbivores. Am. Nat..

[54] Villalba JJ, Burritt EA, Clair SBS (2014). Aspen (*Populus tremuloides* Michx.) intake and preference by mammalian herbivores: the role of plant secondary compounds and nutritional context. J. Chem. Ecol..

[55] Rhodes AC, Larsen RT, Maxwell JD, St. Clair SB (2018). Temporal patterns of ungulate herbivory and phenology of aspen regeneration and defense. Oecologia.

[56] Cingolani AM, Posse G, Collantes MB (2005). Plant functional traits, herbivore selectivity and response to sheep grazing in Patagonian steppe grasslands. J. Appl. Ecol..

[57] Bran D, Ayesa J, Lopez C (2002). Áreas ecológicas de Neuquen.

[58] Mueller J (2001). Producción ovina en Argentina, situación actual y perspectivas futuras. Boletín Inf. INTA.

[59] Aguiar MR, Sala OE (1998). Interactions among grasses, shrubs, and herbivores in Patagonian grass-shrub steppes. Ecol. Austral..

[60] Zamora-Nasca LB, Relva MA, Núñez MA (2019). Ungulate browsing on introduced pines differs between plant communities: implications for invasion process and management. Austral. Ecol..

[61] Bonvissuto GL, Somlo RC, Lanciotti ML, Carteau AG, Busso CA (2008). Guías de Condición para Pastizales Naturales de ‘Precordillera’, ‘Sierras y Mesetas’ y ‘Monte Austral’ de Patagonia.

[62] Siffredi GL (2013). Guía para la evaluación de Pastizales. Para las áreas ecológicas de Sierras y Mesetas Occidentales y de Monte de Patagonia Norte.

[63] SENASA (2015). Manual de Bienestar Animal Un enfoqe práctico para el buen manejo de especies domésticas durante su tenencia, producción, concentración, transporte y faena.

[64] Álvarez, J. M. *et al. Bienestar animal Ovino* (2005).

[65] Mumm R, Hilker M (2006). Direct and indirect chemical defence of pine against folivorous insects. Trends Plant Sci..

[66] Moreira X (2013). Inducibility of chemical defences by two chewing insect herbivores in pine trees is specific to targeted plant tissue, particular herbivore and defensive trait. Phytochemistry.

[67] Crawley, M. J. Mixed-effect models. In *The R Book* 681–714 (Wiley, 2013).

[68] Zuur AF, Ieno EN, Walker NJ, Saveliev AA, Smith GM (2009). Mixed Effects Models and extensions in Ecology with R.

[69] Lenth, M. R. Package ‘lsmeans’. *CRAN* (2013).

[70] Bates DM, Machler M, Bolker B, Walker S (2015). Fitting linear mixed-effects models using lme4. J. Stat. Softw..

[71] Skaug H, Fournier D, Nielsen A, Magnusson A, Bolker BM (2012). Generalized linear mixed models using ‘AD model builder’. Optim. Methods Softw..

[72] Hothorn, T. *et al. Package ‘multcomp’ - Simultaneous Inference in General Parametric Models*. (2017). https://cran.r-project.org/web/packages/multcomp/multcomp.pdf 10.1002/bimj.200810425 18481363

[73] Giraudoux, P., Antonietti, J.-P., Beale, C., Pleydell, D. & Treglia, M. *Package ‘pgirmess’: Spatial Analysis and Data Mining for Field Ecologists* (2018). 10.1145/3097983.3098168doi:

[74] Dormann CF (2013). Collinearity: a review of methods to deal with it and a simulation study evaluating their performance. Ecography (Cop.).

[75] Peuke AD (2010). Correlations in concentrations, xylem and phloem flows, and partitioning of elements and ions in intact plants. A summary and statistical re-evaluation of modelling experiments in Ricinus communis. J. Exp. Bot..

[76] Wickham, H. *ggplot2*. *Elegant Graphics for Data Analysis* (2009). doi:10.1007/978-0-387-98141-3.

[77] R Development Core Team. R: A language and environment for statistical computing. Vienna, Austria. https://www.R-project.org/ (2018).

[78] Zamora R, Gómez JM, Hódar JA, Castro J, García D (2001). Effect of browsing by ungulates on sapling growth of Scots pine in a mediterranean environment: consequences for forest regeneration. For. Ecol. Manag..

[79] Estell RE (1998). Effects of volatile compounds on consumption of alfalfa pellets by sheep. J. Anim. Sci..

[80] Kruska D, Stephan H (1973). Volumenvergleich allokortikaler Hirnzentren hei Wild- und Hausschweinen. Acta Anat..

[81] Arnold GW, De Boer ES, Boundy CAP (1980). The influence of odour and taste on the food preferences and food intake of sheep. Aust. J. Agric. Res..

[82] Tribe DE (1949). The importance of the sense of smell to the grazing sheep. J. Agric. Sci..

[83] Villalba JJ, Provenza FD (1999). Effects of food structure and nutritional quality and animal nutritional state on intake behaviour and food preferences of sheep. Appl. Anim. Behav. Sci..

[84] Evju M, Austrheim G, Halvorsen R, Mysterud A (2009). Grazing responses in herbs in relation to herbivore selectivity and plant traits in an alpine ecosystem. Oecologia.

[85] Cadenasso ML, Pickett STA, Morin PJ (2002). Experimental test of the role of mammalian herbivores on old field succession: Community structure and seedling survival. J. Torrey Bot. Soc..

[86] Capó EA, Aguilar R, Renison D (2016). Livestock reduces juvenile tree growth of alien invasive species with a minimal effect on natives: a field experiment using exclosures. Biol. Invasions.

[87] Dimock EJ, Silen RR, Allen VE (1976). Genetic resistance in Douglas -fir to damage by snowshoe hare and black-tailed deer. For. Sci..

[88] Mobæk R, Mysterud A, Egil Loe L, Holand Ø, Austrheim G (2009). Density dependent and temporal variability in habitat selection by a large herbivore; an experimental approach. Oikos.

[89] Iason GR, O’Reilly-Wapstra JM, Brewer MJ, Summers RW, Moore BD (2011). Do multiple herbivores maintain chemical diversity of Scots pine monoterpenes?. Philos. Trans. R. Soc. Lond. B. Biol. Sci..

