# Supplementary material for: Sheep feeding preference as a tool to control pine invasion in Patagonia: influence of foliar toughness, terpenoids and resin content
Source: Sci Rep. 2020 Jul 21;10:12113. doi: 10.1038/s41598-020-68748-y (PMC7374590; doi:10.1038/s41598-020-68748-y)
Supplement: Supplementary file 1 — Supplementary Information 1. [file 41598_2020_68748_MOESM1_ESM.docx]

Sheep feeding preference as a tool to control pine invasion in Patagonia: influence of foliar toughness, terpenoids and resin content

Lucía B. Zamora-Nasca ^a^, Romina D. Dimarco ^d*^, Daniela Nassini ^c^, Pablo A. Alvear ^a^, Ariel Mayoral ^a^, Martin A. Nuñez ^b^, M. Andrea Relva ^a^

^a^ Instituto de Investigaciones en Biodiversidad y Medio Ambiente, CONICET - Universidad Nacional del Comahue, Bariloche, Río Negro, Argentina

^b^ Grupo Ecología de Invasiones. Instituto de Investigaciones en Biodiversidad y Medio Ambiente, CONICET - Universidad Nacional del Comahue, Bariloche, Río Negro, Argentina

^c^ Centro Atómico Bariloche, CONICET, Bariloche, Río Negro, Argentina

^d^ Grupo de Ecología de Poblaciones de Insectos, IFAB, INTA‐CONICET, Bariloche, Río Negro, Argentina

^*^corresponding author:

Romina D. Dimarco

Grupo de Ecología de Poblaciones de Insectos

IFAB (INTA – CONICET)

Modesta Victoria 4450, (8400), S. C. de Bariloche

Rio Negro, Argentina

Tel: +54 294 442-2731

e-mail: rominadimarco@gmail.com

**Supporting Information**

*Supplementary Methods S1: Estimation of the sheep stocking rate*

To carry on the experiments of preference of herbivory, we exposed seedlings of four species of pines to sheep herbivory inside enclosures. Each enclosure was stocked with sheep at a density typically recommended for the study area (0.2 – 0.3 wethers/ha/year for grassland) ^1,2^. We selected Merino wethers (castrated male sheep) of the same age, all of whom had a similar initial diet with similar nutritional components and all of whom had been exposed to pines before the experiment. The latter feature is important because individuals may develop a preference for one species or another over time ^3,4^.

To estimate the appropriate sheep densities, we used as a basis the pasture guidelines developed by specialists from INTA (Instituto Nacional de Tecnología Agropecuaria, Argentina), which are the standard for the region and are widely used by ranchers ^1,2^. Since these guidelines provided stocking rate recommendations per hectare per year and dry forage production per year, we calculated the area and grazing time corresponding to our experimental design. We took into account that wethers consume 0.920 kg of dry forage daily. The dry forage production per year and per hectare estimated for grassland communities where we set the experiment is 200 - 300 kg. We also considered the vegetation Use Factor (UF), a value corresponding to a proportion of effectively consumable forage that can be grazed by sheep without compromising a sustainable forage production over time ^5^. The UF assumed was 40%. Thus, the calculated stock rate was two wethers in each enclosure for three days.

Summarizing, if daily forage requirement of wethers is 0.920 kg:

- 2 wethers x 3 days= 5.520 kg in 0.0625 ha (25x25m enclosure), in 1 ha this corresponds to 88.32 kg

The vegetation Use Factor assumed was 40%, so 88.32 kg corresponds to 40% UF, the 100% of forage available is 220.8 kg. This last value is between the calculated Dry forage/year available in the grassland with the conditions and vegetation composition like as our study, (200 – 300 kg/ha) ^1^.

We realized that these forage estimations consider the vegetation annual variation, and the herbivore forage consumption along the year and not an instant stock rate like we had applied. However, we believe that this design provides a tool to carry out replicated experiments, allowing the control of numerous influential variables of this process, like the stocking rate, the age and type of herbivore, and surrounding vegetation. Also, we carefully control that there was no overgraze through the consideration of the vegetation Factor Use and doing our experiments in late spring, the season of a major production of forage of the year and with the greatest contribution to the means dry annual forage estimations

**References**

1. Bonvissuto, G. L., Somlo, R. C., Lanciotti, M. L., Carteau, A. G. & Busso, C. A. *Guías de Condición para Pastizales Naturales de ‘Precordillera’, ‘Sierras y Mesetas’ y ‘Monte Austral’ de Patagonia*. (Instituto Nacional de Tecnología Agrpecuaria - INTA, 2008).

2. Siffredi, G. L. *et al.* *Guía para la evaluación de Pastizales. Para las áreas ecológicas de Sierras y Mesetas Occidentales y de Monte de Patagonia Norte*. (INTA, 2013).

3. Squibb, R. C., Provenza, F. D. & Balph, D. F. Effect of age of exposure on consumption of a shrub by sheep. *J. Anim. Sci.* **68**, 987–997 (1990).

4. Walker, J. W., Hemenway, K. G., Hatfield, P. G. & Glimp, H. A. Training lambs to be weed eaters: Studies with leafy spurge. *J. Range Manag.* **45**, 245–249 (1992).

5. Golluscio, R. A. *et al.* Divergencias en la estimación de receptividad ganadera en el noroeste de la Patagonia: diferencias conceptuales y consecuencias prácticas. *Ecol. Austral* **19**, 3–18 (2009).

**Supporting Information**

*Supplementary Tables S2: Post-hoc comparisons* *of analyses of sheep browse preference*

Table S2.1. Results of post-hoc comparisons of herbivory damage between four pines species (Scheffe method).

| **Variable** | **Contrast** | **Estimate** | **ES** | **df** | **z-value** | **p-value** |
| --- | --- | --- | --- | --- | --- | --- |
| **Browsing incidence** | J - C | 0,892 | 0,376 | Inf | 2,367 | **0,049** |
|  | P - C | -0,427 | 0,349 | Inf | -1,222 | 0,469 |
|  | R - C | -1,667 | 0,361 | Inf | -4,614 | **<,0001** |
|  | R - P | -1,239 | 0,349 | Inf | -3,548 | **0,001** |
|  | C - P | 0,427 | 0,349 | Inf | 1,222 | 0,469 |
|  | J - P | 1,319 | 0,377 | Inf | 3,494 | **0,001** |
|  | C - R | 1,667 | 0,361 | Inf | 4,614 | **<,0001** |
|  | J - R | 2,559 | 0,397 | Inf | 6,444 | **<,0001** |
|  | P - R | 1,239 | 0,349 | Inf | 3,548 | **0,001** |
|  | P - J | -1,319 | 0,377 | Inf | -3,495 | **0,001** |
|  | R - J | -2,559 | 0,397 | Inf | -6,444 | **<,0001** |
|  | C - J | -0,892 | 0,376 | Inf | -2,367 | **0,049** |
| **Relative reduction in height** | J - C | -0,601 | 0,124 | Inf | -4,812 | **<,0001** |
|  | P - C | -0,271 | 0,119 | Inf | -2,266 | 0,06 |
|  | R - C | -0,324 | 0,120 | Inf | -2,688 | **0,020** |
|  | R - P | -0,053 | 0,123 | Inf | -0,430 | 0,923 |
|  | C - P | 0,271 | 0,119 | Inf | 2,266 | 0,063 |
|  | J - P | -0,329 | 0,126 | Inf | -2,606 | **0,025** |
|  | C - R | 0,324 | 0,120 | Inf | 2,688 | **0,020** |
|  | J - R | -0,276 | 0,127 | Inf | -2,165 | 0,081 |
|  | P - R | 0,053 | 0,123 | Inf | 0,430 | 0,923 |
|  | P - J | 0,329 | 0,126 | Inf | 2,606 | **0,025** |
|  | R - J | 0,276 | 0,127 | Inf | 2,165 | 0,081 |
|  | C - J | 0,601 | 0,124 | Inf | 4,812 | **<,0001** |
| **Probability of a terminal bud being browsed** | J - C | -1,216 | 0,335 | Inf | -3,623 | **0,001** |
|  | P - C | -0,649 | 0,318 | Inf | -2,040 | 0,108 |
|  | R - C | -0,543 | 0,318 | Inf | -1,709 | 0,214 |
|  | R - P | 0,105 | 0,323 | Inf | 0,326 | 0,956 |
|  | C - P | 0,649 | 0,318 | Inf | 2,040 | 0,108 |
|  | J - P | -0,566 | 0,338 | Inf | -1,673 | 0,229 |
|  | C - R | 0,543 | 0,318 | Inf | 1,709 | 0,214 |
|  | J - R | -0,672 | 0,339 | Inf | -1,980 | 0,123 |
|  | P - R | -0,105 | 0,323 | Inf | -0,326 | 0,956 |
|  | P - J | 0,566 | 0,338 | Inf | 1,673 | 0,229 |
|  | R - J | 0,672 | 0,339 | Inf | 1,980 | 0,123 |
|  | C - J | 1,216 | 0,335 | Inf | 3,623 | **0,001** |
| **Browsing intensity** | J - C | -0,600 | 0,144 | Inf | -4,142 | **0,0001** |
|  | P - C | 0,009 | 0,183 | Inf | 0,054 | 0,998 |
|  | R - C | -0,066 | 0,242 | Inf | -0,273 | 0,969 |
|  | R - P | -0,076 | 0,265 | Inf | -0,288 | 0,966 |
|  | C - P | -0,009 | 0,183 | Inf | -0,054 | 0,998 |
|  | J - P | -0,610 | 0,180 | Inf | -3,374 | **0,002** |
|  | C - R | 0,066 | 0,242 | Inf | 0,273 | 0,969 |
|  | J - R | -0,533 | 0,240 | Inf | -2,221 | 0,070 |
|  | P - R | 0,076 | 0,265 | Inf | 0,288 | 0,966 |
|  | P - J | 0,610 | 0,180 | Inf | 3,374 | **0,002** |
|  | R - J | 0,533 | 0,2403 | Inf | 2,221 | 0,070 |
|  | C - J | 0,600 | 0,144 | Inf | 4,142 | **0,0001** |
| **Probability of a seedling being defoliated** | J - C | 2,406 | 0,385 | Inf | 6,245 | **<,0001** |
|  | P - C | 1,312 | 0,385 | Inf | 3,407 | **0,001** |
|  | R - C | -1,913 | 0,780 | Inf | -2,451 | **0,039** |
|  | R - P | -3,225 | 0,750 | Inf | -4,299 | **0,0001** |
|  | C - P | -1,312 | 0,385 | Inf | -3,407 | **0,001** |
|  | J - P | 1,093 | 0,310 | Inf | 3,519 | **0,001** |
|  | C - R | 1,913 | 0,780 | Inf | 2,451 | **0,0393** |
|  | J - R | 4,319 | 0,751 | Inf | 5,749 | **<,0001** |
|  | P - R | 3,225 | 0,750 | Inf | 4,298 | **0,0001** |
|  | P - J | -1,093 | 0,310 | Inf | -3,519 | **0,0013** |
|  | R - J | -4,319 | 0,751 | Inf | -5,749 | **<,0001** |
|  | C - J | -2,406 | 0,385 | Inf | -6,245 | **<,0001** |
| **Probability of survival** | J - C | 2,314 | 1,049 | Inf | 2,206 | 0,073 |
|  | P - C | -0,333 | 0,467 | Inf | -0,714 | 0,788 |
|  | R - C | -0,160 | 0,484 | Inf | -0,332 | 0,954 |
|  | R - P | 0,173 | 0,456 | Inf | 0,379 | 0,940 |
|  | C - P | 0,333 | 0,467 | Inf | 0,714 | 0,788 |
|  | J - P | 2,648 | 1,036 | Inf | 2,554 | **0,029** |
|  | C - R | 0,160 | 0,484 | Inf | 0,332 | 0,954 |
|  | J - R | 2,475 | 1,044 | Inf | 2,369 | **0,048** |
|  | P - R | -0,173 | 0,456 | Inf | -0,379 | 0,940 |
|  | P - J | -2,648 | 1,036 | Inf | -2,554 | **0,029** |
|  | R - J | -2,475 | 1,044 | Inf | -2,370 | **0,048** |
|  | C - J | -2,314 | 1,049 | Inf | -2,206 | 0,073 |

**Note:** C: *P. contorta*. P: *P. ponderosa*. R: *P. radiata*. J: *P. jeffreyi.*

**Supporting Information**

*Supplementary Tables S3: Post-hoc comparisons* *of analyses of anti-herbivores chemical compounds and physical traits*

Table S3.1. Results of post-hoc comparisons of Total monoterpenes between four pines species.

| **Variable** | **Contrast** | **Estimate** | **SE** | **t value** | **Pr(>\|t\|)** |
| --- | --- | --- | --- | --- | --- |
| **Total monoterpenes** | P - C == 0 | 1.21 | 0.44 | 2.761 | 0.0357 * |
|  | R - C == 0 | -2.77 | 0.44 | -6.306 | <0.001 *** |
|  | J - C == 0 | -0.05 | 0.44 | -0.136 | 0.9991 |
|  | R - P ==0 | -3.99 | 0.44 | -9.068 | <0.001 *** |
|  | J - P == 0 | -1.27 | 0.44 | -2.897 | 0.0247 * |
|  | J - R == 0 | 2.71 | 0.44 | 6.17 | <0.001 *** |

**Note:** C: *P. contorta*. P: *P. ponderosa*. R: *P. radiata*. J: *P. jeffreyi.* Significances: 0 ‘***’ 0.001 ‘**’ 0.01 ‘*’ 0.05 ‘.’ 0.1 ‘ ’ 1

Table S3.2. Results of post-hoc comparisons of α-pinene, β-pinene, 3-carene, β -phellandrene, non-volatile resin content, water content and needle toughness between four pines species.

| **Variable** | **Contrast** | **obs.dif** | **critical.dif** | **difference** |
| --- | --- | --- | --- | --- |
| **Resin content** | C-P | 22.66 | 16.82 | TRUE |
|  | C-R | 20.93 | 16.82 | TRUE |
|  | C-J | 1.06 | 16.82 | FALSE |
|  | P-R | 43.60 | 16.82 | TRUE |
|  | P-J | 23.73 | 16.82 | TRUE |
|  | R-J | 19.86 | 16.82 | TRUE |
| **α -pinene** | C-P | 25.75 | 19.38 | TRUE |
|  | C-R | 0.55 | 19.38 | FALSE |
|  | C-J | 48.40 | 19.38 | TRUE |
|  | P-R | 26.30 | 19.38 | TRUE |
|  | P-J | 22.65 | 19.38 | TRUE |
|  | R-J | 48.95 | 19.38 | TRUE |
| **β -pinene** | C-P | 21.80 | 19.38 | TRUE |
|  | C-R | 18.37 | 19.38 | FALSE |
|  | C-J | 14.22 | 19.38 | FALSE |
|  | P-R | 40.17 | 19.38 | TRUE |
|  | P-J | 36.02 | 19.38 | TRUE |
|  | R-J | 4.15 | 19.38 | FALSE |
| **3-carene** | C-P | 25.15 | 19.38 | TRUE |
|  | C-R | 19.62 | 19.38 | TRUE |
|  | C-J | 18.32 | 19.38 | FALSE |
|  | P-R | 44.77 | 19.38 | TRUE |
|  | P-J | 43.47 | 19.38 | TRUE |
|  | R-J | 1.30 | 19.38 | FALSE |
| **β -phellandrene** | C-P | 45.83 | 19.38 | TRUE |
|  | C-R | 41.68 | 19.38 | TRUE |
|  | C-J | 28.30 | 19.38 | TRUE |
|  | P-R | 4.15 | 19.38 | FALSE |
|  | P-J | 17.53 | 19.38 | FALSE |
|  | R-J | 13.38 | 19.38 | FALSE |
| **Needle toughness** | C-P | 25.48 | 19.38 | TRUE |
|  | C-R | 18.00 | 19.38 | FALSE |
|  | C-J | 36.53 | 19.38 | TRUE |
|  | P-R | 43.48 | 19.38 | TRUE |
|  | P-J | 11.05 | 19.38 | FALSE |
|  | R-J | 54.53 | 19.38 | TRUE |
| **Water content** | C-P | 29.33 | 16.82 | TRUE |
|  | C-R | 13.67 | 16.82 | FALSE |
|  | C-J | 15.67 | 16.82 | FALSE |
|  | P-R | 15.67 | 16.82 | FALSE |
|  | P-J | 45.0 | 16.82 | TRUE |
|  | R-J | 29.33 | 16.82 | TRUE |

**Note:** C: *P. contorta*. P: *P. ponderosa*. R: *P. radiata*. J: *P. jeffreyi.*
